# SELF-REPORTED VERSUS DIAGNOSIS-BASED DISEASED LIFE EXPECTANCY AMONG OLDER COLOMBIANS

**DOI:** 10.1093/geroni/igad104.1245

**Published:** 2023-12-21

**Authors:** Margarita Maria Osuna, Eileen Crimmins, Jennifer Ailshire

**Affiliations:** University of Southern California Leonard Davis School of Gerontology, Los Angeles, California, United States; University of Southern California, Los Angeles, California, United States; University of Southern California, Los Angeles, California, United States

## Abstract

The number of older adults in Colombia will soon double and it is essential to understand the disease burden in this population. Disease prevalence is often estimated from medical diagnoses, which can underestimate disease in populations with less health care access and low health literacy. We compare differences in diseased life expectancy (DLE) using self-reported vs. measured markers for diabetes and hypertension using data from the 2015 health and wellbeing survey of older adults in Colombia (SABE-Col), a nationally representative survey of Colombians ages 60+. Respondents reported whether they had ever been told by a doctor that they had either diabetes or hypertension. Using direct measures of glucose and blood pressure and clinical cut-points, we determined which respondents were considered to have diabetes or hypertension. We used the Sullivan method to estimate life expectancy with and without disease. Data from self-reports alone generally suggest lower DLE than from both self-reported and measured disease statuses. At age 60, women have a total life expectancy of 20.56 years. We find a lower DLE for hypertension using self-reported data (12.66 years) than measured (13.24 years). Conversely, reported diabetes is similar (4.18 years) to the measured (4.28 years) DLE. Men, at age 60, have a total life expectancy of 18.04 years. DLE for hypertension using self-reports is lower (8.78 years) than measured (9.61 years). Similarly, DLE for diabetes is lower for self-report (2.98 years) than measured (3.04). Our findings show the burden of disease in this rapidly aging population.

